# Acute retinal necrosis caused by co-infection with multiple viruses in a natalizumab-treated patient: a case report and brief review of literature

**DOI:** 10.1186/s12886-021-02096-x

**Published:** 2021-09-16

**Authors:** Kasra Cheraqpour, Aliasghar Ahmadraji, Ali Rashidinia, Maziyar Irannejad, Mansoor Shahriari

**Affiliations:** 1 Eye Research Center, Farabi Eye Hospital, Tehran University of Medical Sciences, Qazvin Square, 1336616351 Tehran, Iran; 2 Imam Hossein Medical Center, Shahid Beheshti University of Medical Sciences, Tehran, Iran

**Keywords:** Acute retinal necrosis, Herpes simplex virus, Varicella-zoster virus, Multiple sclerosis, Natalizumab

## Abstract

**Background:**

Acute retinal necrosis is considered a rare infectious uveitis. This condition is usually caused by varicella-zoster virus or herpes simplex virus. Acute retinal necrosis caused by co-infection with multiple viruses is extremely rare. Herein, we report a case of acute retinal necrosis caused by co-infection with herpes simplex virus (type I and II) and varicella-zoster virus (VZV) in a natalizumab-treated patient due to multiple sclerosis.

**Case presentation:**

An adult man presented with a complaint of decreased vision of the right eye from 12 days ago. He was a known case of multiple sclerosis receiving natalizumab. Examination of the right eye revealed severe conjunctival injection, fine diffuse keratic precipitates, 3 + anterior chamber and vitreous cells, elevated intraocular pressure (26 mmHg), a blurred optic disk with hemorrhagic patches, and occlusive vasculitis plus confluent necrotizing patches in the peripheral retina compatible with diagnosis of acute retinal necrosis. He underwent anterior chamber and vitreous tap, and real-time PCR detected HSV I & II and VZV on the vitreous specimen. A second PCR showed the same result. After neurological consultation, natalizumab was discontinued and intravenous acyclovir was started followed by oral acyclovir and oral prednisolone to control the disease, which was successful.

**Conclusions:**

Although rare, multiple-viral infection should be considered in the physiopathology of acute retinal necrosis, especially in immunosuppressed patients.

## Background

Acute retinal necrosis (ARN) is a rare infectious uveitis usually caused by some members of the herpes virus family [1]. In 2007, a study reported an incidence rate of 1 in 1.6 to 2 million per year in the UK [2]. ARN may occur in both immunocompetent and immunosuppressed individuals. There is no sexual predilection and most of the cases occur between the fifth and seventh decades of life [3]. About two thirds of the patients experience unilateral involvement whereas the disease may spread to the fellow eye within 1–6 weeks in one third of the cases. Bilateral occurrence of ARN is more common in neonates and immunosuppressed patients [3]. The complications of ARN include retinal detachment (RD), macular ischemia, and optic atrophy [4]. Although the diagnosis of ARN is clinical, polymerase chain reaction (PCR), as a highly specific method, is routinely performed on intra-ocular fluid specimens in suspected cases [5].

ARN caused by multiple viruses is an extremely rare finding. Herein, we describe a case of acute retinal necrosis caused by co-infection with herpes simplex virus (type I and II) and varicella-zoster virus (VZV) in a natalizumab-treated patient due to multiple sclerosis.

## Case presentation

**Fig. 1:**
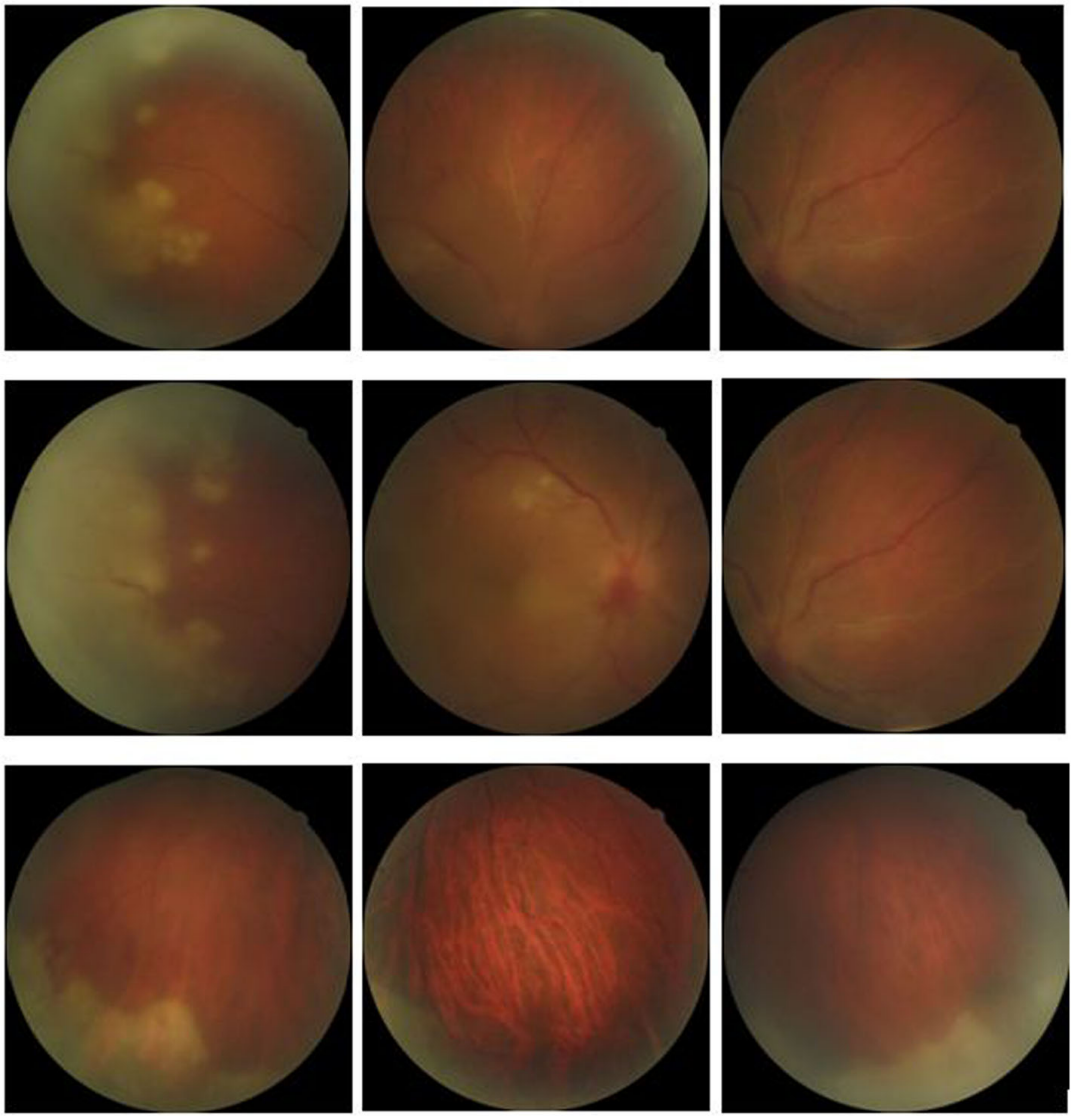
Fundus photograph of the right eye showing hazy media due to vitritis, a blurred optic disk with hemorrhagic patches, occlusive vasculitis, and confluent necrotizing patches in the peripheral retina

A 54-year-old man presented to the Emergency Department of Farabi Eye Hospital with a complaint of decreased vision of the right eye from 12 days ago. His past medical history was positive for multiple sclerosis (MS). He was a current user of natalizumab since 3 years ago. At presentation, the best-corrected visual acuity (BCVA) of the left eye was 20/20 and slit-lamp examination and funduscopy revealed no pathologic findings in the left eye. The right eye had a BCVA of 20/200, severe conjunctival injection, fine diffuse keratic precipitates (KPs), 3 + anterior chamber (AC) and vitreous cells, elevated intraocular pressure (26 mmHg), a blurred optic disk with hemorrhagic patches, and occlusive vasculitis plus confluent necrotizing patches in the peripheral retina (shown in Fig. 1). According to the clinical findings, ARN was the most possible scenario. The patient was scheduled for immediate AC and vitreous tap, and PCR detected HSV type I, HSV type II, and VZV on vitreous samples. Target DNA was isolated and genotyped using real-time PCR with Taqman™(ABI®, USA) and hybridization probe (Roche®, Germany) in a private laboratory. To exclude the possibility of the lab error or contamination, PCR was rechecked, which showed the same result. After neurological consult, natalizumab was discontinued. Intravenous acyclovir (10 mg/kg every 8 h for 1 week) was used in the induction phase of the treatment. Response to treatment was significant and inflammatory signs started to resolve. Oral acyclovir was used as an adjunct to oral prednisolone on a tapering strategy for 3 months as the maintenance phase. In addition, 360°prophylactic laser photocoagulation was done to prevent subsequent RRD. However, our attempt was not successful and the patient developed refractory RRD for which he underwent pars plana vitrectomy, silicone oil injection, and endolaser photocoagulation for multiple times. Despite all of the procedures, the right eye did not gain a BCVA better than hand motions (HM).

## Discussion and conclusions

Acute retinal necrosis (ARN) is a sight-threatening uveitis commonly caused by varicella-zoster virus (VZV), herpes simplex virus (type I and type II), cytomegalovirus (CMV), and Epstein-barr virus (EBV) [5]. In addition, a case of ARN caused by human adenovirus has been reported recently [5]. Both immunocompetent and immunosuppressed individuals may develop ARN. Thus, the characteristics of the causative agent may be more determining than the status of host’s immune system [6]. The differential diagnosis list of this entity includes other causes of infectious uveitis such as progressive outer retinal necrosis (PORN), CMV retinitis, toxoplasmosis, and syphilis as well as non-infectious conditions like intraocular lymphoma and Behcet disease [7]. Recent herpes virus infection, genetic factors, and immunosuppression may prone the patients to ARN [1]. In addition to corticosteroids, monoclonal antibodies such as natalizumab can be considered a predisposing factor for developing ARN as in our case [1].

Natalizumab, which is indicated for the treatment of several conditions such as Crohn’s disease and relapsing multiple sclerosis, is a recombinant humanized monoclonal antibody [8]. This drug prevents the migration of WBCs to the CNS and GI tract. Hence, the CD4+/CD8 + ratio is lower in the cerebrospinal fluid of patients using natalizumab compared to the peripheral blood. Such interactions may increase the vulnerability to opportunistic infections. Studies have shown an association between herpes family viruses and CNS and eye infections in natalizumab-treated patients [8, 9]. Discontinuation of natalizumab is recommended in such patients [1]. To the best of our knowledge few reports of retinal necrosis in natalizumab-treated patients are available in the literature. Table 1 provides a brief review on the related reports.

**Table 1:** Brief review on available reports of retinal necrosis in natalalizumab-treated patients (*M *male, *F* female, *OD* right eye, *OS* left eye, *OU* both eyes, *N/A* not applicable, *CSF* cerebrospinal fluid, *NLP* no light perception, *PR* present report)

No. [Ref]	Age Sex	Time of Diagnosis of MS	Natalizumab regimen	Time of diagnosis of ARN	Involvement	Initial vision	Diagnostic sampling	Antiviral treatment	Final vision
1 [1]	34 F	2011	Monthly infusion since 2012 in which the last infusion was 3 weeks before the symptoms.	2015	Bilateral ARN	-OD: 20/25 -OS: 20/50	Aqueous/VZV	Intravitreal injections of foscarnet and ganciclovir and intravenous acyclovir	-OD: 20/30 at -OS: HM (combined tractional and rhegmatogenous retinal detachment)
2 [10]	49 N/A	2006	Monthly infusions since 2007	2012	Combined retinal (OU) and CNS vasculitis	N/A	CSF/VZV	Intravenous acyclovir + plasma exchange therapy	NLP
3 [11]	46 M	2001	Start since 2006	2018	OS	0.2	CSF/VZV	Intravenous acyclovir and intravitreal ganciclovir injections	0.5
4 [12]	51 M	N/A	N/A	N/A	OD	20/30	Serology/Positive IgG and IgM for VZV	Oral valacyclovir	20/30
5 [13]	54 F	N/A	N/A	N/A	OS (progressive outer retinal necrosis)	20/125	Aqueous/VZV	Intravenous and intravitreal antivirals	20/125
6 [14]	37 F	2004	Start since 2007	2017	Combined retinal (OS) and CNS vasculitis	0.2	Aqueous/VZV	Intravenous acyclovir and intravitreal foscarnet	0.16
	59 F	1995	Start since 2006	2019	Combined retinal (OU) and CNS vasculitis	NLP	Aqueous/VZV	Intravenous acyclovir and intravitreal ganciclovir	NLP
7 [PR]	54 M	2015	Monthly infusion since 2017 in which the last infusion was 2 weeks before the symptoms.	2020	OD	20/200	Vitreous/VZV, HSV I, HSV II	Intravenous acyclovir	HM (rhegmatogenous retinal detachment)

Diagnosis of ARN is clinical. Common clinical features include a remarkable inflammatory reaction of both anterior chamber and vitreous, at least one focus of retinal necrosis in the peripheral retina with circumferential spread, rapid progression in the lack of appropriate treatment, and an arterial occlusive vasculopathy [5]. A well-defined, smooth, geographic border separates necrotic retina from healthy parts. In addition, scant areas of retinal hemorrhage may be present [6]. Despite a clinical-based diagnosis, PCR of intraocular fluids is routinely used in practice to identify the causing virus [15].

To the best of our knowledge, this is one of the very rare reports of ARN due to multiple viral infections. Shida Chen et al. reported a 52-year-old man with a diagnosis of acute retinal necrosis caused by multiple viruses [16]. They used two primer pairs for samples from diagnostic vitrectomy; the first one could detect the presence of HSV type I, HSV type II, EBV, and CMV and the second one could identify VZV, human herpes virus 6 (HHV-6), and HHV-7. Since EBV and CMV are not common causes of ARN and HHV-6 and HHV-7 are not discussed in pathogenesis of ARN, the authors concluded that the most probable condition was co-infection with HSV and VZV [16]. Tomoko Nakamura et al. published another similar case report in 2015 [17]. They identified VZV and CMV DNA in the vitreous, tear, saliva, and skin of an old healthy woman. However, they concluded that CMV was not responsible for ARN in this woman according to clinical characteristics and response to acyclovir. Unlike these two reports, the PCR results of our case showed co-infection with three viruses (HSV type I and type II, and VZV), which can be considered a unique aspect of the present report. We believe that immunosuppression secondary to natalizumab was the underlying reason for infection with multiple viruses. It should be mentioned real-time PCR is highly reproducible, rapid, sensitive and specific technique [18]. Nevertheless, this technique was performed on the specimens for two times to exclude the possibility of lab error. Although the reported case was interesting and rare, it seems that multiple viral infections did not affect the response to treatment in our case. Therefore, we believe this finding has research values more than clinical implications.

Historically intravenous acyclovir or oral valacyclovir are the most common agents for induction phase of treatment and single use of these drugs has been reported in remarkable number of studies. However, adjunction of intravitreal antiviral agents has been emerged as a more popular option in the recent years due to reduction in the duration of hospitalization, vision loss, and retinal detachment [19]. The induction phase of the treatment in our case was started with intravenous acyclovir, which was successful. Ganciclovir and foscarnet are expensive and poorly available in our center. So these drugs are used only for our severe and refractory cases but not in routine approach.

In conclusion, although rare, infection with multiple viruses should be considered in the physiopathology of acute retinal necrosis, especially in immunosuppressed patients.

## Data Availability

The data is available from the corresponding author on reasonable request.

## Abbreviations

ARN: Acute retinal necrosis
RD: Retinal detachment
PCR: Polymerase chain reaction
HSV: Herpes simplex virus
VZV: Varicella-zoster virus
MS: Multiple sclerosis
BCVA: Best-corrected visual acuity
KP: Keratic precipitate
AC: Anterior chamber
RRD: Rhegmatogenous retinal detachment
CMV: Cytomegalovirus
EBV: Epstein-barr virus
HHV: Human herpes virus
